# Towards a common terminology: a simplified framework of interventions to promote and integrate evidence into health practices, systems, and policies

**DOI:** 10.1186/1748-5908-9-51

**Published:** 2014-05-01

**Authors:** Heather Colquhoun, Jennifer Leeman, Susan Michie, Cynthia Lokker, Peter Bragge, Susanne Hempel, K Ann McKibbon, Gjalt-Jorn Y Peters, Kathleen R Stevens, Michael G Wilson, Jeremy Grimshaw

**Affiliations:** 1 Clinical Epidemiology Program, Ottawa Hospital Research Institute. Ottawa Hospital – General Campus, 501 Smyth Road, C.P. 711, K1H 8 L6 Ottawa, ON, Canada; 2 School of Nursing, University of North Carolina at Chapel Hill, Chapel Hill, NC, USA; 3 Research Department of Clinical, Educational, and Health Psychology, University College London, 1-19 Torrington Place, WC1E 7HB, London, UK; 4 Department of Clinical Epidemiology and Biostatistics, Health Information Research Unit, McMaster University, CRL Building, 1280 Main Street West, L8S 4 K1, Hamilton, ON, Canada; 5 National Trauma Research Institute, Monash University and The Alfred Hospital, Level 4, 89 Commercial Road, 3004 Melbourne, VIC, Australia; 6 RAND Corporation, 1776 Main Street, m4339, Santa Monica 90407, CA, USA; 7 Methology & Statistics of the Faculty of Psychology, Open University of the Netherlands, P.O. box 2960, 6401 DL Heerlen, The Netherlands; 8 Academic Center for Evidence-Based Practice, University of Texas Health Science Center, San Antonio, 7703 Floyd Curl Drive, 78229-3900 San Antonio, TX, USA; 9 Department of Clinical Epidemiology and Biostatistics, and The McMaster Health Forum, Centre for Health Economics and Policy Analysis, McMaster University, CRL 223, 1280 Main Street West, L8S 4 K1, Hamilton, ON, Canada; 10 Department of Medicine, University of Ottawa, Ottawa Hospital – General Campus, 501 Smyth Road, C.P. 711, K1H 8 L6 Ottawa, ON, Canada

**Keywords:** Knowledge translation, Implementation science, Classification, Consensus, Dissemination, Implementation

## Abstract

**Background:**

A wide range of diverse and inconsistent terminology exists in the field of knowledge translation. This limits the conduct of evidence syntheses, impedes communication and collaboration, and undermines knowledge translation of research findings in diverse settings. Improving uniformity of terminology could help address these challenges. In 2012, we convened an international working group to explore the idea of developing a common terminology and an overarching framework for knowledge translation interventions.

**Findings:**

Methods included identifying and summarizing existing frameworks, mapping together a subset of those frameworks, and convening a multi-disciplinary group to begin working toward consensus. The group considered four potential approaches to creating a simplified framework: melding existing taxonomies, creating a framework of intervention mechanisms rather than intervention strategies, using a consensus process to expand one of the existing models/frameworks used by the group, or developing a new consensus framework.

**Conclusions:**

The work group elected to draft a new, simplified consensus framework of interventions to promote and integrate evidence into health practices, systems and policies. The framework will include four key components: strategies and techniques (active ingredients), how they function (causal mechanisms), how they are delivered (mode of delivery), and what they aim to change (intended targets). The draft framework needs to be further developed by feedback and consultation with the research community and tested for usefulness through application and evaluation.

## Background

In many respects, the most troublesome problems of any science centre around its most basic terms and fundamental concepts, and not around its more sophisticated concerns. Indeed to the extent that everything either follows from or is based on a discipline’s most basic terms and fundamental concepts, problems at a higher level can always be traced back to problems at a more fundamental level. (Mitroff & Sagasti, 1973) [1].

Efforts to build the science of how to most effectively promote and support the use of evidence in health and healthcare policy and practice have been variably termed ‘knowledge translation (KT)’, ‘implementation science’, ‘quality improvement’, ‘dissemination’, etc. Within each of these fields of study, researchers have developed a variety of terms for their approaches and interventions. For example, in an analysis of the titles and abstracts of over 20,000 quality improvement publications, Walshe found that authors used numerous different terms to present an essentially similar set of approaches, with terms changing in frequency of use over time [2]. Similarly, in an effort to develop an inventory of KT-related terms, McKibbon *et al*. identified 100 different terms to describe KT research [3].

This diversity and inconsistency of terminology is a potential barrier to synthesizing, advancing, and applying the findings from what we will refer to as knowledge translation. The KT field is in the early stages of development and as yet lacks shared conceptualizations of problems, potential solutions, and a common language. This makes it difficult for researchers to learn from each other’s work; to collaborate across geographic boundaries, disciplines and sectors; or to search for and synthesize findings from KT research [3].

Examples of how inconsistent terminology can impede advancement are numerous. McKibbon and colleagues attempted unsuccessfully to develop a search filter specific to KT [4]; only 46 of 100 KT terms were found in titles or abstract of KT articles [3]. Systematic reviews on KT interventions consistently conclude that variability in intervention reporting impeded the synthesis [5-7].

An additional problem is the variety of models, frameworks and taxonomies that have been developed to guide intervention design and evaluation. A recent review of models and frameworks for dissemination and implementation suggests that at least 61 such models exist [8]. Whilst the diversity reflects their development in different contexts for different purposes, it potentially limits effective communication between research and implementation groups and risks introducing inefficiencies into efforts to interpret and accumulate evidence and to apply evidence to improvements in practice and policy. Given this, there is a need to try to develop shared frameworks and terminologies or, at least, one overarching framework that researchers might apply to understand and communicate about each other’s frameworks and terminologies.

Working towards consensus about terms used in the field was an objective of a Canadian Institutes of Health Research multi-site grant (FRN#88368) awarded through KT Canada. KT Canada is a network of Canadian experts in KT with goals to improve how research results are communicated; to develop a consensus on KT terminology and methods for measuring success; to evaluate KT approaches; and to find ways to ensure that KT efforts have a lasting impact across the continuum of care. During the course of this work, we became aware of other international researchers who were involved in or were proposing similar terminology programs of research, and our ambition was to explore whether it would be possible to consolidate the work of these other individuals and groups. Hence, in 2012, members of the KT Canada project team (JG, AM, CL, HC) convened an international workgroup to explore the idea of developing a common language and an overarching framework for KT interventions. By interventions, we mean activities intended to increase KT at the level of practice, systems and policies. While there is a need to examine the broad range of terminology issues present in the field of KT, an initial focus on KT interventions was deemed a useful starting point given the complexities often inherent in KT intervention design [9].

Members of the team reviewed the literature and queried other experts in the field to identify participants, and 35 researchers and information specialists from the fields of behavioral science, health systems research, policy, nursing, quality improvement, medicine, public health, rehabilitation, and library science from Canada, the UK, USA, the Netherlands, and Australia were invited to participate, and expressed support and interest, in the project. A total of 12 of these invitees attended a two-day meeting in Canada in September 2012 (see Table 1 for a list of attendees, their group affiliation and research program focus). The general aim was to clarify terminology for KT interventions with the goal of improving evidence searching and synthesis and communication between research groups, disciplines and countries, thereby increasing the profile of KT in scientific and other arenas. This paper presents methods used to move towards the groups’ aims, outcomes of the meeting including an initial framework for discussion and debate, and future development plans.

**Table 1:** **Additional file participants attending a two**-**day meeting in Ottawa**, **ON**, **Canada**, **in Sept. 2012**

**Name**	**Group**	**Focus**
Peter Bragge	National Trauma Research Institute, Monash University and Alfred Hospital, Australia	Quality Improvement/Evidence Synthesis
Mike Wilson	McMaster University, Centre for Health Economics and Policy Analysis, Canada	Policy
Ann McKibbon Cynthia Lokker	McMaster University, Health Information Research Unit, Canada	Information retrieval
Susanne Hempel	RAND Corporation, California, USA	Quality improvement
Susan Michie	University College London, UK	Behaviour change
Jennifer Leeman	University of North Carolina, USA	Public health
Jeremy Grimshaw Heather Colquhoun	Ottawa Hospital Research Institute and University of Ottawa, Canada	Provider behaviour change
Kathleen Stevens	University of Texas San Antonio, USA	Patient safety
Gjalt-Jorn Peters	Open University, The Netherlands	Behaviour change

### Methods used to achieve aims

### Describing and applying frameworks for KT interventions

In preparation for the meeting, the 35 invitees were asked to electronically share frameworks they had developed or commonly used in their work promoting and integrating evidence into practice. Documents provided an overarching conceptualization of the field of KT, outlining stages and/or components of a process or system [10-14], or included lists of terms and their definitions [15,16]. A number of frameworks were developed to characterize different types of interventions; some described interventions to change individual behaviors [17,18], while others targeted change at the level of organizational systems and infrastructures and public policies [19-21].

As an exercise, a group of five meeting attendees mapped four intervention frameworks and taxonomies that were known and familiar to the group – The Behaviour Change Wheel [21], The Cochrane Effective Practice and Organization of Care (EPOC) framework [22], the Leeman Taxomony [23], and the Behaviour Change Technique Taxomony v1 [24] – into a single framework. The goal was to attempt a parsimonious and comprehensive common platform to which other terms could be mapped. Although the mapping exercise did not result in a unified framework, it illustrated the key issues and challenges and served as a starting point for future work (see Additional file 1 for the mapping attempt).

### Methods to develop a framework

During the meeting, participants presented their experiences with the frameworks that they had used in their work, reviewed the mapping exercise, and discussed how to develop a framework that KT scholars might use to understand and communicate about each other’s intervention frameworks and terminologies. The intent was not to suggest that the frameworks that participants used should be abandoned in favour of a new model, but rather to develop a framework that would function as a ‘terminology facilitator’ – an overarching framework of interventions that specified standardized terms for sharing, reporting, and communicating across the field. Four potential approaches to creating a simplified framework were considered:

1. Meld existing taxonomies. The mapping exercise highlighted several obstacles to melding existing taxonomies: the terms used within taxonomies overlap and the relationships among them are unclear; terms within the taxonomies are not consistent in scope, scale or function, with some terms describing broad approaches to implementation (*e.g*., quality improvement) and others describing more discrete strategies (*e.g*., reminder systems).

2. Create a framework of intervention mechanisms. Following the recommendation that interventions be characterized by their mechanisms rather than their components [25], we discussed three approaches to modeling intervention mechanisms: the Theoretical Domains Framework [17,18], Intervention Mapping [26], and the Behaviour Change Wheel [21]. By ‘intervention mechanisms,’ we mean the processes or mediators by which an intervention effects change. The workgroup acknowledged the value that this work has contributed to advancing intervention design. However, creating one framework based on mechanisms was thought to be unhelpful due to the breadth of strategies and techniques used in KT interventions.

3. Use a consensus process to expand one of the existing frameworks used by the group. This type of consensus was considered not to be feasible on the basis of a review of two studies of definitions of quality improvement (QI) interventions. The first, using a process of consultation with expert panels, developed a definition of QI but was not successful in applying that definition to synthesize QI literature [27]. A follow-up study aimed to develop definitional features of continuous quality improvement (CQI) [28] but found that subjective interpretation of constructs, difficulty measuring constructs, heterogeneity in published papers, and poor reporting of QI prevented the achievement of consensus on classifying QI interventions [28].

4. Develop a new consensus framework. The workgroup agreed on the option of developing a new consensus framework of KT interventions that would guide the development of a standardized vocabulary and the development of common language. Several principles were used to guide the discussion, informed by guidance from Dr. Stuart Nelson, then Head of the Medical Subject Headings Section of the US National Library of Medicine, and based on his experiences with indexing and evolving language and scientific fields. These included: clarify the shared purpose first; consider the framework a first draft; use language accessible for all sectors of KT, focus on developing a simple high-level standard language for interventions; involve users and get feedback. The group agreed to the following shared objective: ‘To work towards a simplified framework of interventions to promote and integrate evidence into health practices, systems, and policies’.

### Meeting outcome – draft simplified model

The framework categorizes interventions according to four elements that form the basis of the interventions and the specific terms used to describe them. While it is recognized as a first attempt, the aim for an eventual framework would be to incorporate a standardized vocabulary that any KT scholar could use. Potentially, the framework could function as a guide to frame or ‘think about’ interventions as well as a way to understand and describe causal pathways for intervention effectiveness. The four elements include intervention strategies and techniques (active ingredients), how they function (causal mechanisms), how they are delivered (mode of delivery), and what they aim to change (intended targets).

1. Active ingredients are the components that have the capacity to bring about change and are defining characteristics of interventions [29,30]. Active ingredients can be categorized broadly, as is done in the interventions and policy levels of the Behavior Change Wheel [21], or characterized at the level of specific strategies or techniques [24]. Most active ingredients target determinants of behavior, which may be in the domains of motivation (*e.g*., attitude or self-efficacy), capability (*e.g*., skills) or opportunity (*e.g*., environmental barriers) [26].

2. Causal mechanisms are the processes or mediators by which an intervention effects change. We looked to the Behavior Change Wheel as a starting point for identifying the causal mechanisms of interventions. This identifies nine functions by which interventions affect outcomes (*e.g*., education, persuasion, incentivisation). An intervention’s causal mechanisms may vary across different phases of the intervention process. This may be particularly true for policy and systems-level changes, which often require engaging stakeholders, collaboratively formulating the policy or plan, and then persuading decision-makers to support and enact it. Only then can the actual implementation of the policy or plan begin [31]. The relative importance of different causal mechanisms is influenced by a variety of contextual factors.

3. Mode of delivery [29] or practical application [26] refers to the way in which an active ingredient is applied. How intervention components are delivered or applied (*e.g*., face to face, brochure, mass media) needs to be distinguished from the active ingredients they embody. Because the active ingredients concern generic psychological, organizational, and regulatory processes, a generically defined active ingredient can be delivered in a variety of applications or media. We need to build our knowledge about what modes of delivery work best for what active ingredients for what purposes.

4. Intended target includes the intervention’s intended effects and beneficiaries. Intervention targets have been categorized as: change behavior of individuals; change coordinated behaviors among multiple staff; change policies, procedures, and technologies; increase organizational or system-level capacity or improve infrastructure; create or strengthen collaborative partnerships or coalitions; and change systems [32].

This framework of interventions can be mapped onto existing taxonomies/terminologies as a next step in developing and refining the framework. An example of undertaking this step would be to map each element in the simplified framework to the concepts in existing intervention frameworks to test the validity of or refine the framework. Some authors test and refine their taxonomy/model by selecting relevant research studies and categorizing the intervention according to the taxonomy (*e.g*., [33]). This is an iterative process whereby items are added or refined to ensure that all interventions can be described. We recognize that the goal of a simplified framework of interventions to promote and integrate evidence into health practices, systems and policies might not be achievable or even desirable. However, the framework could be one approach to assisting KT scholars from diverse perspectives to develop common understandings.

Recommendations for solving issues of terminology have included using expertise from taxonomic fields, standardizing vocabulary and definitions, and advocating the adoption of a small, common set of terms [3]. We have attempted these steps. While representation of the participants included QI, patient safety, public health, behaviour change, policy, information retrieval, and taxonomy, the group was limited to 12 participants. Our aim in going forward is to have a larger group with broader representation in terms of background and geography. Input and feedback from potential users of the framework is sought; those interested in participating should contact the corresponding author.

## Conclusion

We summarize the work of an international working group that investigated the possibility of a consensus on terminology used to describe and categorize interventions. The framework presented here is a draft to encourage debate and reflection and hopefully move in the direction of a consensus about the desirability of, and method for, developing a framework that is fit for purpose. It is an empirical question as to whether this will be useful for the understanding and use of terms and their application in academic, clinical, public health and policy settings.

## Abbreviations

KT: Knowledge translation; QI: Quality improvement; CQI: Continuous quality improvement.

## Competing interests

Susan Michie is an associate editor and Jeremy Grimshaw is an Editorial Board member for Implementation Science. Neither was involved in the editorial process for this paper and decisions regarding this manuscript were made independently by other Implementation Science editors.

## Author’s contributions

HC, JL, SM, and JG drafted the manuscript. CL provided editorial support. All authors were involved in developing the simplified model and read, provided edits, and approved the final manuscript.

## Supplementary Material

Additional file 1 The mapping of: Behaviour Change Wheel, EPOC categories, Leeman taxonomy and behaviour change techniques. Click here for file
