# Erratum: Towards a common terminology: a simplified framework of interventions to promote and integrate evidence into health practices, systems, and policies

**DOI:** 10.1186/s13012-014-0154-4

**Published:** 2014-12-21

**Authors:** Heather Colquhoun, Jennifer Leeman, Susan Michie, Cynthia Lokker, Peter Bragge, Susanne Hempel, K Ann McKibbon, Gjalt-Jorn Y Peters, Kathleen Stevens, Michael G Wilson, Jeremy Grimshaw

**Affiliations:** Clinical Epidemiology Program, Ottawa Hospital Research Institute. Ottawa Hospital – General Campus, 501 Smyth Road, C.P. 711, Ottawa, ON K1H 8 L6 Canada; School of Nursing, University of North Carolina at Chapel Hill, Chapel Hill, NC USA; Research Dept of Clinical, Educational, and Health Psychology, University College London, 1-19 Torrington Place, London, WC1E 7HB UK; Dept of Clinical Epidemiology and Biostatistics, Health Information Research Unit, McMaster University, CRL Building, 1280 Main Street West, Hamilton, ON L8S 4 K1 Canada; National Trauma Research Institute, Monash University and The Alfred Hospital, Level 4, 89 Commercial Road, Melbourne, Victoria 3004 Australia; RAND Corporation, 1776 Main Street, m4339, Santa Monica, CA 90407 USA; Methology & Statistics of the Faculty of Psychology, Open University of the Netherlands, PO box 2960, 6401 DL Heerlen, The Netherlands; Academic Center for Evidence-Based Practice, University of Texas Health Science Center, San Antonio, 7703 Floyd Curl Drive, San Antonio, TX 78229-3900 USA; Department of Clinical Epidemiology and Biostatistics, and The McMaster Health Forum, Centre for Health Economics and Policy Analysis, McMaster University, CRL 223, 1280 Main Street West, Hamilton, ON L8S 4 K1 Canada; Department of Medicine, University of Ottawa, Ottawa Hospital – General Campus, 501 Smyth Road, C.P. 711, Ottawa, ON K1H 8 L6 Canada

## Correction

In the course of type-setting the article [1], the first two sentences of the background section were inadvertently printed without quotation marks. These two sentences are a direct quote from the paper referenced, and as such, require quotation marks. In addition, the formatting for this reference was printed with the number [1] as well as the author names. Correct formatting includes only the [1]. The referencing for these sentences is now correct. The authors regret this error.

The erroneous text was:

In many respects, the most troublesome problems of any science centre around its most basic terms and fundamental concepts, and not around its more sophisticated concerns. Indeed to the extent that everything either follows from or is based on a discipline’s most basic terms and fundamental concepts, problems at a higher level can always be traced back to problems at a more fundamental level. (Mitroff & Sagasti, 1973) [1].

This text should have read:

“In many respects, the most troublesome problems of any science centre around its most basic terms and fundamental concepts, and not around its more sophisticated concerns. Indeed to the extent that everything either follows from or is based on a discipline’s most basic terms and fundamental concepts, problems at a higher level can always be traced back to problems at a more fundamental level” [1] (page 117).
