# Supplementary material for: Towards a common terminology: a simplified framework of interventions to promote and integrate evidence into health practices, systems, and policies
Source: Implement Sci. 2014 May 1;9:51. doi: 10.1186/1748-5908-9-51 (PMC4021969; doi:10.1186/1748-5908-9-51)
Supplement: Additional file 1 — The mapping of: Behaviour Change Wheel, EPOC categories, Leeman taxonomy and behaviour change techniques. [file 1748-5908-9-51-S1.docx]

**Additional File 1. The mapping of: Behaviour Change Wheel [1], EPOC categories [2], Leeman taxonomy [3] and behaviour change techniques [4].**

|  | **Behaviour Change Wheel [1]** *Intervention function 1-9/ Policy category 10-16* | **EPOC category [2]** | **Leeman taxonomy [3]** | **Behaviour change techniques [4]** |
| --- | --- | --- | --- | --- |
| 1 | Education:  *Increasing knowledge or understanding* | 2.1.1 *Professional interventions*  a) Distribution of educational materials (Distribution of published or printed recommendations for clinical care, including clinical practice guidelines, audio-visual materials and electronic publications. The materials may have been delivered personally or through mass mailings.)  b) Educational meetings (Health care providers who have participated in conferences, lectures, workshops or traineeships.)  d) Educational outreach visits (Use of a trained person who met with providers in their practice settings to give information with the intent of changing the provider’s practice. The information given may have included feedback on the performance of the provider(s). | Education  Raising awareness | Feedback on the behaviour  Feedback on the outcome(s) of the behaviour  Biofeedback  Self-monitoring of behaviour  Self-monitoring of outcome of behaviour  Cue signaling reward  Satiation  Prompts/cue  Information about antecedents  Re-attribution  Behavioural experiments  Information about social and environmental consequences  Information about health consequences  Information about emotional consequences  Information about others’ approval |
| 2 | Persuasion: *Using communication to induce positive or negative feelings or stimulate action* | 2.1.1 *Professional interventions*  c) Local consensus processes (Inclusion of participating providers in discussion to ensure that they agreed that the chosen clinical problem was important and the approach to managing the problem was appropriate.)  e) Local opinion leaders (Use of providers nominated by their colleagues as ‘educationally influential’. The investigators must have explicitly stated that their colleagues identified the opinion leaders.) | Persuasion via interpersonal channels & via reinforcing beliefs  Opinion leader | Feedback on the behaviour  Feedback on the outcome(s) of the behaviour  Biofeedback  Re-attribution  Focus on past success  Verbal persuasion about capability  Persuasive source  Framing/reframing  Identity associated with changed behaviour  Identification of self as role model  Information about social and environmental consequences  Information about health consequences  Information about emotional consequences  Salience of consequences  Information about others’ approval  Social comparison |
| 3 | Incentivisation: *Creating expectation of reward* | 2.1.2 *Financial interventions*  2.1.2.1 *Provider interventions*  a) Fee-for-service (provider has been paid for number and type of service delivered)  b) Prepaid (no other description)  c) Capitation (provider was paid a set amount per patient for providing  specific care)  d) Provider salaried service (provider received basic salary for providing  specific care)  e) Prospective payment (provider was paid a fixed amount for health care in  advance)  f) Provider incentives (provider received direct or indirect financial reward or benefit for doing specific action)  g) Institution incentives (institution or group of providers received direct or indirect financial rewards or benefits for doing specific action)  h) Provider grant/allowance (provider received direct or indirect financial reward or benefit not tied to specific action)  i) Institution grant/allowance (institution or group of providers received  direct or indirect financial reward or benefit not tied to specific action)  l) Formulary (added to reimbursable available products) | Financial incentives  Reinforcing expectations of positive outcomes | Paradoxical instructions  Feedback on the behaviour  Feedback on the outcome(s) of the behaviour  Biofeedback  Self-monitoring of behaviour  Self-monitoring of outcome of behaviour  Monitoring of behaviour by others without evidence of feedback  Monitoring outcome of behaviour by others without evidence of feedback  Cue signalling reward  Remove aversive stimulus  Reward approximation  Rewarding completion  Situation-specify reward  Reward incompatible behaviour  Reduce reward frequency  Reward alternate behaviour  Remove punishment  Social reward  Material reward  Material reward (outcome)  Self-reward  Non-specific reward  Incentive  Behavioural contract  Commitment  Discrepancy between current behaviour and goal  Imaginary reward |
| 4 | Coercion: *Creating expectation of punishment or cost* | 2.1.2 *Financial interventions*  2.1.2.1 *Provider interventions*  j) Provider penalty (provider received direct or indirect financial penalty for  inappropriate behaviour)  k) Institution penalty (institution or group of providers received direct or indirect financial penalty for inappropriate behaviour)  l) Formulary (removed from reimbursable available products) |  | Feedback on the behaviour  Feedback on the outcome(s) of the behaviour  Biofeedback  Self-monitoring of behaviour  Self-monitoring of outcome of behaviour  Monitoring of behaviour by others without evidence of feedback  Monitoring outcome of behaviour by others without evidence of feedback  Remove access to the reward  Punishment  Behaviour cost  Remove reward  Future punishment  Behavioural contract  Commitment  Discrepancy between current behaviour and goal  Incompatible beliefs  Anticipated regret  Imaginary punishment |
| 5 | Training: *Imparting skills* |  |  |  |
| 6 | Restriction: *Using rules to reduce the opportunity to engage in the target behaviour (or to increase the target behaviour by reducing the opportunity to engage in competing behaviours)* |  |  |  |
| 7 | Environmental restructuring: *Changing the physical or social context* | 2.1.3.3 *Structural interventions*  a) Changes to the setting/site of service delivery (e.g. moving a family planning service from a hospital to a school)  b) Changes in physical structure, facilities and equipment (e.g change of location of nursing stations, inclusion of equipment where technology in question is used in a wide range of problems and is not disease specific, for example an MRI scanner.)  c) Changes in medical records systems (e.g. changing from paper to computerised records, patient tracking systems)  2.1.1 *Professional interventions*  h) Reminders (Patient or encounter specific information, provided verbally, on paper or on a computer screen, which is designed or intended to prompt a health professional to recall information. This would usually be encountered through their general education; in the medical records or through interactions with peers, and so remind them to perform or avoid  some action to aid individual patient care. Computer aided decision  support and drugs dosage are included.) | Modified medical record system  Environment-al change  Changes to the work environment  Reminder systems  Interpersonal networks and communica-tion | Cue signalling reward  Remove access to the reward  Remove aversive stimulus  Satiation  Exposure  Associative learning  Reduce prompt/cue  Prompts/cue  Adding objects to the environment  Restructuring the physical environment  Restructuring the social environment |
| 8 | Modelling: *Providing an example for people to aspire to or imitate* |  | Opinion leader |  |
| 9 | Enablement: *Increasing means/reducing barriers to increase capability or opportunity (beyond education, training and environmental restructuring)* | 2.1.1 *Professional interventions*  f) Patient mediated interventions (New clinical information (not previously available) collected directly from patients and given to the provider e.g. depression scores from an instrument.)  g) Audit and feedback (Any summary of clinical performance of health care over a specified period of time. The summary may also have included recommendations for clinical action. The information may have been obtained from medical records, computerised databases, or observations from patients.)  h) Reminders (Patient or encounter specific information, provided verbally, on paper or on a computer screen, which is designed or intended to prompt a health professional to recall information. This would usually be encountered through their general education; in the medical records or through interactions with peers, and so remind them to perform or avoid  some action to aid individual patient care. Computer aided decision  support and drugs dosage are included.)  2.1.2 *Financial interventions*  2.1.2.2 *Patient interventions*  a) Premium (Patient payment for health insurance. It is important to determine if the patient paid the entire premium, or if the patient’semployer paid some of it. This includes different types of insurance plans.)  b) Co-payment (Patient payment at the time of health care delivery in addition to health insurance e.g. in many insurance plans that cover prescription medications the patient may pay 5 dollars per prescription, with the rest covered by insurance.)  c) User-fee (Patient payment at the time of health care delivery.)  d) Patient incentives (Patient received direct or indirect financial reward or benefit for doing or encouraging them to do specific action.)  e) Patient grant/allowance (Patient received direct or indirect financial reward or benefit not tied to specific action.)  f) Patient penalty (Patient received direct or indirect financial penalty for specified behaviour e.g. reimbursement limits on prescriptions.)  2.1.3 *Organisational interventions*  2.1.3.2 *Patient orientated interventions*  a) Mail order pharmacies (e.g. compared to traditional pharmacies)  b) Presence and functioning of adequate mechanisms for dealing with patients’ suggestions and complaints  c) Consumer participation in governance of health care organisation | External change agent  Guidance from manager  Audit and feedback  Performance evaluations  Workgroup develops change  Reminder systems  Workgroup oversight  Pilot testing  Designation of a change leader | Social support (unspecified)  Social support (practical)  Social support (emotional)  Reduce negative emotions  Conserve mental resources  Pharmacological support  Self-monitoring of behaviour  Self-monitoring of outcome of behaviour  Behaviour substitution  Overcorrection  Generalisation of a target behaviour  Graded tasks  Avoidance/reducing exposure to cues for the behaviour  Adding objects to the environment  Restructuring the physical environment  Restructuring the social environment  Distraction  Body changes  Behavioural experiments  Mental rehearsal of successful performance  Focus on past success  Self-talk  Verbal persuasion about capability  Self-reward  Goal setting (behaviour)  Goal setting (outcome)  Behavioural contract  Commitment  Action planning  Review behaviour goal(s)  Review outcome goal(s)  Discrepancy between current behaviour and goal  Problem solving  Pros and cons  Comparative imagining of future outcomes  Valued self-identity  Framing/reframing  Incompatible beliefs  Identity associated with changed behaviour  Identification of self as role model  Salience of consequences  Monitoring of emotional consequences  Anticipated regret  Imaginary punishment  Imaginary reward  Vicarious consequences |
| 10 | Communication/marketing: *Using print, electronic, telephonic or broadcast media* | i) Marketing (Use of personal interviewing, group discussion (‘focus groups’), or a survey of targeted providers to identify barriers to change and subsequent design of an intervention that addresses identified barriers.)  j) Mass media ((i) varied use of communication that reached great numbers of people including television, radio, newspapers, posters, leaflets, and booklets, alone or in conjunction with other interventions; (ii) targeted at the population level.) |  | See map of policy category to intervention functions |
| 11 | Guidelines: *Creating documents that recommend or mandate practice. This includes all changes to service provision* |  |  |  |
| 12 | Fiscal: *Using the tax system to reduce or increase the financial cost* |  |  |  |
| 13 | Regulation: *Establishing rules or principles of behaviour or practice* | 2.1.4 *Regulatory interventions*  Any intervention that aims to change health services delivery or costs by regulation or law. (These interventions may overlap with organisational and financial interventions.)  a) Changes in medical liability  b) Management of patient complaints  c) Peer review  d) Licensure |  | See map of policy category to intervention functions |
| 14 | Legislation: *Making or changing laws* |  |  |  |
| 15 | Environmental/ social planning: *Designing and/or controlling the physical or social environment* |  |  |  |
| 16 | Service provision: *Delivering a service* | 2.1.3.3 *Structural interventions*  d) Changes in scope and nature of benefits and services  e) Presence and organisation of quality monitoring mechanisms  f) Ownership, accreditation, and affiliation status of hospitals and other facilities  g) Staff organisation  2.1.3.1 *Provider orientated interventions*  a) Revision of professional roles (Also known as ‘professional substitution’, ‘boundary encroachment’ and includes the shifting of roles among health professionals. For example, nurse midwives providing obstetrical care; pharmacists providing drug counselling that was formerly provided by nurses and physicians; nutritionists providing nursing care; physical therapists providing nursing care. Also includes expansion of role to include new tasks.)  b) Clinical multidisciplinary teams (creation of a new team of health  professionals of different disciplines or additions of new members to the team who work together to care for patients)  c) Formal integration of services (bringing together of services across sectors or teams or the organisation of services to bring all services together at one time also sometimes called ‘seamless care’)  d) Skill mix changes (changes in numbers, types or qualifications of staff)  e) Continuity of care (including one or many episodes of care for inpatients or outpatients)  • Arrangements for follow-up.  • Case management (including co-ordination of assessment, treatment and arrangement for referrals)  g) Communication and case discussion between distant health professionals (e.g. telephone links; telemedicine; there is a television/video link between specialist and remote nurse practitioners) | Centralised care management | See map of policy category to intervention functions |

*The following were excluded because an outcome rather than an intervention: EPOC category: f) Satisfaction of providers with the conditions of work and the material and psychic rewards (e.g. interventions to ‘boost morale’)

Reference List

1. Michie S, van Stralen MM, West R: **The behaviour change wheel: A new method for characterising and designing behaviour change interventions.** *Implement Sci* 2011, **6:** 42.

2. Cochrane Effective Practice and Organisation of Care Group. **EPOC resources for review authors.** http://epoc.cochrane.org/ . 2010.

3. Leeman J, Baernholdt M, Sandelowski M: **Developing a theory-based taxonomy of methods for implementing change in practice.** *J Adv Nurs* 2007, **58:** 191-200.

4. Michie S, Richardson M, Johnston M, Abraham C, Francis JJ, Hardeman W *et al*.: **The Behavior Change Technique Taxonomy (v1) of 93 hierarchically-clustered techniques: building an international consensus for the reporting of behavior change interventions.**  *Ann Behav Med*  2013, **46:** 81-95.
